# Interest in linkage to PrEP among people who inject drugs accessing syringe services; Miami, Florida

**DOI:** 10.1371/journal.pone.0231424

**Published:** 2020-04-16

**Authors:** Young Jo, Tyler S. Bartholomew, Susanne Doblecki-Lewis, Allan Rodriguez, David W. Forrest, Jasmine Tomita-Barber, Juan Oves, Hansel E. Tookes

**Affiliations:** 1 Department of Public Health Sciences, Miller School of Medicine, University of Miami, Miami, Florida, United States of America; 2 Division of Infectious Diseases, Department of Medicine, Miller School of Medicine, University of Miami, Miami, Florida, United States of America; 3 Department of Anthropology, College of Arts and Sciences, University of Miami, Miami, Florida, United States of America; 4 Herbert Wertheim College of Medicine, Florida International University, Miami, Florida, United States of America; University of Cyprus, CYPRUS

## Abstract

**Background:**

People who inject drugs (PWID) are at an increased risk for HIV infection due to injection and sexual risk behaviors. This study aims to examine PrEP knowledge, awareness, and willingness to be linked to PrEP services at a syringe services program (SSP), and examine the relationship between substance use and interest in PrEP linkage.

**Methods:**

Data were collected using a cross-sectional survey of IDEA SSP clients in Miami, FL (N = 157). Based on reported substance injected, participants were classified into opioid-only injection or polysubstance injection. Socio-demographics and HIV risk were examined using Pearson’s Chi-Squared analysis. Bivariate and multivariable logistic regression models were used to test for significant correlates of interest in PrEP linkage.

**Results:**

Only 28.3% of PWID surveyed had previously heard of PrEP. However, 57.2% were interested in receiving more information about PrEP. In the adjusted model, people with opioid-only use were significantly less likely to report interest in being linked to PrEP.

**Conclusion:**

Knowledge, awareness, and interest in being linked to PrEP were low among PWID surveyed. No participants of the study were successfully linked to PrEP services through direct referrals. Further research is needed to examine low threshold service delivery of PrEP to PWID at SSPs.

## Introduction

There is clear evidence that sharing needles and syringes is a direct route for transmission of HIV—1 in 10 new HIV infections in the United States is among people who inject drugs (PWID) [1]. In addition to injection risk, PWID more frequently experience sexual coercion, sexual violence, and condomless sex, increasing risk of sexual transmission of HIV [2, 3]. A culture of sharing injection equipment and high risk sexual behaviors results in PWID’s having 22 times the risk of HIV infection compared to the population overall [4]. Although research on syringe services programs (SSPs) has shown effectiveness in reducing HIV infections [5] and risky injection behavior [6], social and structural factors act as barriers to prevention and treatment of HIV in PWID. Drug control policies such as the 1970 Controlled Substances Act have contributed to an increase in mass incarceration and the establishment of social stigma among people who use drugs [7]. Furthermore, intransigent stigma and mistrust of the health system play a critical role in preventing PWID from accessing social and health services such as treatment for substance use disorder.

Research has shown that SSP users are more likely to enroll in drug treatment services compared to non-SSP users [8]. SSPs provide a window of opportunity for a series of harm reduction interventions among PWID through the exchange of new syringes as well as provision of health services and education, including pre-exposure prophylaxis (PrEP). PrEP, approved by the FDA in 2012, is a once daily pill that reduces risk of acquiring HIV by over 90% [9]. This public health strategy has shown to be effective in reducing HIV risk, and SSPs may also be ideal centers for fostering PrEP awareness and access [10–12]. Willingness of PWID to use PrEP has been established in other settings, with 35–63% of PWID indicating willingness to take PrEP in diverse cohorts [10, 13–16]. Variables independently associated with willingness to use PrEP include younger age, no regular employment, sex work, multiple recent sexual partners, and requiring help injecting, suggesting that PrEP in combination with other forms of harm reduction could be especially helpful for PWID [14].

Awareness of PrEP among PWID, however, is low, with only 3% of respondents in a Vancouver survey reporting prior knowledge of PrEP [14]. Another study examined awareness of PrEP among women who inject drugs and found that women who had a discussion about HIV prevention at an SSP were over seven times more likely to be aware of PrEP [17]. Additionally, Roth et al (2018) recently reported that 86% of PWID surveyed preferred to attend an SSP for PrEP instead of a traditional sexually transmitted infection clinic [18]. Access to medical services, including PrEP, is frequently difficult among uninsured individuals with low incomes and structural barriers to care, including lack of Medicaid expansion and other safety-net medical services [19]. Navigation services or integration of PrEP at an SSP could be beneficial in overcoming disparities to PrEP access among socioeconomically disadvantaged populations at risk for HIV, including PWID.

SSPs are already integrated into networks of PWID and deliver a multitude of services designed to reduce the incidence of infectious diseases while promoting harm reduction. In order to reach the current Centers for Disease Control and Prevention goal of Ending the HIV Epidemic, increasing provision of PrEP to PWID in low barrier settings could decrease incidence of HIV in this vulnerable population [17, 18]. While extensive research has been conducted on the effectiveness of PrEP in men who have sex with men, PrEP has been understudied and underutilized among PWID. More importantly, seen in places such as Scott County, Miami, and Seattle, HIV outbreaks among PWID have been occurring more frequently, and substances being injected have differing HIV risk profiles due to associated injection and sexual behavior [20–22]. This current study aims to characterize the difference in PrEP awareness, PrEP knowledge, and interest in PrEP linkage among PWID accessing an SSP.

## Materials and methods

### Ethics

This study was approved by the Institutional Review Board of the University of Miami (IRB# 20160931). Written informed consent was obtained from each participant.

### Study setting

This study was conducted at the pilot IDEA SSP, Florida’s first legal SSP, operated at the University of Miami Miller School of Medicine. The program offers exchange of needles, HIV and hepatitis C testing, naloxone distribution, patient navigation to healthcare services and on site wound care.

### Recruitment

We recruited a convenience sample of participants from the IDEA SSP in Miami, Florida in 2017. Eligibility included 1) 18 years of age or older 2) participant in the SSP 3) non-reactive HIV rapid test at the SSP and 4) ability to provide informed consent. Participants were compensated $25 for completing the survey.

### Measures

#### PrEP variables

We used seven PrEP variables to assess awareness, knowledge, and perceptions about PrEP, including interest in being linked to PrEP services. The study began with a brief explanation of PrEP as a medication people who are HIV negative take to prevent them from getting HIV if they are exposed to it. PrEP awareness was measured by asking participants “Before this study, have your heard about PrEP? (yes/no). All participants were then informed that “PrEP is a pill that is approved to be taken every day to prevent HIV infection. It is for people at risk of HIV infection through sex or drugs.” Knowledge was measured by asking if participants “How effective do you think PrEP is for preventing HIV infection?” A 5-point Likert scale from “not at all effective” to “completely effective” was used to assess this measure. PrEP interest was measured by asking participants “Are you interested in getting linkage to PrEP? (yes/no). Perceptions around PrEP were examined using the following questions: “Would you like to receive information about PrEP?” And “Would you encourage your HIV negative partners to use PrEP, in order to prevent HIV transmission?” In addition, participants were asked what were the main reasons why people may not take PrEP. Participants provided yes and no answers to the following categories: they don’t know about it, they don’t think they are at risk for HIV infection, concern about side effects, cost of the medication, difficult to find a medical provider, they don’t believe it works, and worried about what others would think.

#### Socio-demographics

Socio-demographic, HIV risk, overdose, and drug use data were pulled from each participant’s baseline behavioral assessment administered at enrollment into the SSP. Socio-demographic variables examined included: gender (male/female), race/ethnicity (Non-Hispanic White, Non-Hispanic Black, Hispanic), education level (less than high school/greater than high school), annual income ($0–14,999/greater than $15,000), health insurance (no insurance/Medicaid/private), currently homeless (yes/no), and sexual orientation (gay or bisexual/heterosexual).

#### HIV risk and overdose

Participants were assessed for both injection-related and sexual risk. Participants reported their sharing of injection equipment in the previous 30 days (yes/no). Participants reported whether they reused syringes (≥50% of the time/<50% of the time). Participants were asked how many times they inject, on average, per day in the previous 30 days (less than daily, 1–2, 3–4, 5–7, 8–10, 11–15, and >15). These responses were used to create a dichotomous variable (≤5 injections/>5 injections). The injection cut-point was determined based on equal distribution between the two categories. Participants reported their most frequent location of injection that was categorized into private (at home), public building/restroom, and street/park/public space. Participants were asked if they had unprotected sex in the previous 30 days (yes/no). If they reported yes, they were asked how many sexual partners they had and if they had sex with a person who injects drugs (yes/no). If a participant responded “no” for having sex in the previous 30 days, they were categorized as “0” sexual partners and “no” for having sex with a person who injects drugs. Participants reported ever overdosing (yes/no).

#### Drug group

Participants were asked what drugs had been injected in the previous 30 days. Participants had the opportunity to answer yes/no to the following six drugs: heroin, fentanyl, cocaine, crack, methamphetamine, and speedball (a mix between cocaine and heroin). Based on these responses, participants were categorized into “opioid-only use” (those reporting either heroin or fentanyl injection with no other drugs) and “polysubstance use” (those who reported injecting two or more drugs which could include opioids, cocaine, and methamphetamine). The majority of those reporting polysubstance use reported using cocaine and heroin.

#### Linkage to PrEP protocol

For linkage to PrEP, the interviewer collected participant contact information including phone or email address. Over the course of three months, participants were contacted periodically by email or phone or routinely at the IDEA SSP during exchanges to attempt to facilitate the appointment. At each participant contact, the Florida Department of Health PrEP Clinic was called to help facilitate passive referral. A log of all participant and PrEP Clinic contacts or attempted contacts was kept.

### Data analysis

The original sample surveyed in this study included 159 SSP participants, and no calculation regarding sample size was done prior to analysis. The sample was then stratified by drug injection class (opioid use vs. polysubstance use). Seven participants were excluded due to missing drug use information, creating a final sample of 152 that was analyzed. We compared the differences between opioid and polysubstance use on socio-demographics, HIV risk behaviors and PrEP awareness, knowledge, interest, and perceptions using Pearson’s chi-squared tests. We used bivariate and logistic regression models to examine the association between demographic and risk behavior variables on interest in being linked to PrEP. In addition, a multivariable logistic regression model was used to estimate the adjusted effects of socio-demographics, injection and sexual risk behaviors and drug injection group on interest in being linked to PrEP. All analyses were performed using SAS University statistical software (Version 9.4; SAS Institute, Cary, NC), and all tests were performed at a significance level of 0.05.

## Results

### Socio-demographic and HIV risk behaviors by drug injection group

Table 1 summarizes the socio-demographic, injection and sexual risk behaviors, and overdose by drug injection group. Those who were categorized as polysubstance use were significantly more likely to be currently homeless (65.6% vs. 42.5%, p = 0.008), report sharing injection equipment (45.5% vs. 27.9%, p = 0.025), report injecting in a public building/restroom (24.2% vs. 10.5%, p = 0.023), report injecting in the street, park, or public space (49.2% vs. 34.9%, p<0.001), and report ever overdosing (74.6% vs. 54.3%, p = 0.015). In addition, the polysubstance use group was significantly less likely to have Medicaid (7.1% vs. 19.5%, p = 0.035) compared to the opioid use group.

**Table 1:** Socio-demographics, injection risk, and sexual risk of SSP clients in Miami, FL, by drug injection group.

Characteristic	Opioid-only Use (N = 86)	Polysubstance Use (N = 66)	p-value
	Total N (%)	Total N (%)	
**Age (mean, SD)**	39.7 ± 8.6	37.1 ± 12.3	0.15
**Gender**			0.31
Male	63 (73.3)	53 (80.3)	
Female	23 (26.7)	13 (19.7)	
**Race/Ethnicity**			0.15
Non-Hispanic White	45 (52.3)	36 (59)	
Non-Hispanic Black	5 (5.8)	0 (0)	
Hispanic	36 (41.9)	25 (41)	
**Education Level**			0.18
<High School/GED	37 (43.5)	36 (54.6)	
≥High School/GED	48 (56.5)	30 (45.4)	
**Income (annual)**			0.64
$0–14,999	37 (50.7)	35 (54.7)	
>$15,000	36 (49.3)	29 (45.3)	
**Insurance**			
No insurance	48 (62.3)	46 (82.1)	0.04*
Medicaid	15 (19.5)	4 (7.1)	
Private	14 (18.2)	6 (10.7)	
**Currently Homeless**	31 (42.5)	40 (65.6)	<0.01*
**Sexual Orientation**			0.27
Straight/heterosexual	82 (95.4)	60 (90.9)	
Gay/lesbian/bisexual	4 (4.6)	6 (9.1)	
**Share injection equipment (e.g. syringes, cottons, cookers)**			0.03*
Yes	24 (27.9)	30 (45.5)	
No	62 (72.1)	36 (54.5)	
**Reused syringes**			0.68
<50% of the time	15 (20.3)	11 (17.5)	
≥50% of the time	59 (79.7)	52 (82.5)	
**Number of injections per day**			0.58
≤5 injections	44 (53.0)	32 (48.5)	
>5 injections	39 (47.0)	34 (51.5)	
**Injection Location**			
Private Home	40 (46.5)	26 (39.4)	0.38
Public Building/Restroom	9 (10.5)	16 (24.2)	0.02*
Street, Park, or public space	30 (34.9)	41 (62.1)	<0.01*
**Unprotected Sex**	29 (34.5)	32 (49.2)	0.07
**Number of Sex partners (mean, 95% CI)**	0.73 (0.37, 1.09)	2.41 (0.47, 4.34)	0.06
**Sex with PWID**	21 (24.4)	25 (37.9)	0.07
**Ever Overdosed**	38 (54.3)	47 (74.6)	0.02*

*represents p-value <0.05

### PrEP knowledge and awareness

Only 28.3% of the participants had previously heard of PrEP before the study (Table 2). However, the majority (57.2%) of the entire sample were interested in receiving information about PrEP. In addition, 61.2% believed that PrEP was very to completely effective at preventing HIV, and 89.5% reported they would encourage their HIV negative partners to use PrEP. For reported reasons why people at risk for HIV would not be interested in PrEP, the majority (52.0%) said it was due to lack of knowledge about PrEP and 39.5% reported it was due to cost of medication.

**Table 2:** PrEP awareness, knowledge, and interest among SSP clients in Miami, FL.

PrEP questions	N (%)
***Before this study*, *have your heard about PrEP*?**	
Yes	43 (28.3)
No	109 (71.7)
***Would you like to receive information about PrEP*?**	
Yes	87 (57.2)
No	65 (42.8)
***In what way(s) would you prefer to receive more information about PrEP*?**	
Brochures	72 (47.4)
Videos	20 (13.2)
***How effective do you think PrEP is for preventing HIV infection*?**	
Not at all effective	2 (1.3)
Slightly effective	12 (7.9)
Somewhat effective	43 (28.3)
Very effective	59 (38.8)
Completely effective	34 (22.4)
***Would you encourage your HIV negative partners to use PrEP*, *in order to prevent HIV transmission*?**	
Yes	136 (89.5)
No	7 (4.6)
Do not know	9 (5.9)
***What do you think is the main reason why people at risk of getting infected with HIV would not be interested in PrEP*?**	
They don’t know about it	79 (52.0)
They don’t think they are at risk for HIV infection	34 (22.4)
Concern about side effects	44 (28.9)
Cost of medication	60 (39.5)
Difficult to find a medical provider	30 (19.7)
They don’t believe it works	25 (16.4)
Worried about what others would think	7 (4.6)

### Bivariate and multivariable associations of interest in being linked to prep

Bivariate analysis revealed that the opioid class had decreased odds (OR = 0.44, 95% CI: 0.20, 0.98) of reporting interest in being linked to PrEP. When controlling for age, sex, education level, annual income, insurance, housing status, sexual orientation, condomless sex and sharing injection equipment, the opioid use group had a lower adjusted odds (aOR = 0.35, 95% CI: 0.13, 0.91) of reporting interest in being linked to PrEP (Table 3).

**Table 3:** Unadjusted and adjusted odds of expressing interest in being linked to PrEP among PWID in Miami, FL.

Characteristic	OR	95% CI	aOR	95% CI
**Age (continuous)**	0.98	0.94, 1.01	0.98	0.94, 1.03
**Gender**				
Male	0.58	0.25, 1.33	0.53	0.20, 1.45
Female	Ref	Ref	ref	ref
**Insurance Status**				
Medicaid	1.12	0.36, 3.44	1.41	0.41, 4.85
Private	0.49	0.13, 1.82	0.12	0.01, 1.13
No Insurance	Ref	ref	ref	ref
**Education Level**				
<High School/GED	1.81	0.83, 3.91	2.27	0.90, 5.74
≥High School/GED	Ref	ref	ref	ref
**Income (annual)**				
$0–14,999	0.99	0.47, 2.10	1.37	0.51, 3.64
>$15,000	Ref	ref	ref	ref
**Currently Homeless**	0.84	0.38, 1.87	0.51	0.19, 1.38
**Sexual Orientation**				
Gay/lesbian/bisexual	2.10	0.58, 7.64	2.54	0.48, 13.47
Straight/heterosexual	Ref	ref	ref	ref
**Unprotected Sex in last 30 days**				
Yes	1.44	0.67, 3.11	1.67	0.67, 4.15
No	Ref	ref	ref	ref
**Sharing injection works in last 30 days**				
Yes	0.73	0.32, 1.65	0.60	0.22, 1.58
No	Ref	ref	ref	ref
**Drug Injection Group**				
Opiate-only	0.44*	0.20, 0.98	0.35*	0.13, 0.91
Polysubstance	Ref	ref	ref	ref

*represents p-value <0.05

### PrEP cascade

**Fig 1:**
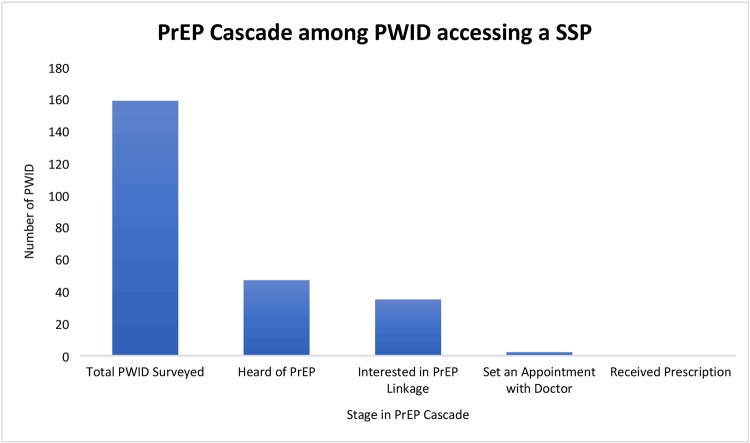
PrEP Cascade among PWID accessing SSP in Miami, FL.

Overall, 43 (28.3%) of the sample had heard of PrEP before the study and 35 (23.0%) expressed interest in being linked to PrEP (Fig 1). Of those expressing interest in being linked to PrEP at the IDEA SSP, only 2 (5.7%) requested doctor’s appointments with a PrEP provider. No participant in the study attended their appointment and successfully received a prescription for PrEP.

## Discussion

Similar to a recent study among PWID in Baltimore [15], the majority of the PWID surveyed in Miami had never heard of PrEP. However, our findings indicate that the majority of the PWID surveyed were interested in hearing about PrEP. Unfortunately, none of the participants in our study were successful in obtaining a PrEP prescription, suggesting that the SSP could improve HIV prevention and overall harm reduction by co-location of PrEP services. Co-location of low-barrier services at an SSP is important because successful PrEP navigation involves having a phone, a calendar, proper identification, an address, transportation to a clinic, and, especially for PWID, a stigma-free environment. While SSPs have been shown to decrease syringe sharing [23], PWID remain at increased risk of contracting HIV due to sexual exposure. Implementation of PrEP services within an SSP has potential to mitigate this risk and enhance HIV prevention efforts among PWID.

Like the recently described HIV outbreak among PWID experiencing homelessness in Seattle, Washington, a recent investigation of an outbreak of HIV among PWID in Miami suggested that there was a link between sexual and injection-related HIV risk [20, 21]. Several recent reports indicate the growing prevalence of sex work as a component of money-generating strategies among PWID [24, 25], and as a risk factor for HIV infection [26–28]. In the outbreak in Scott County, Indiana, transactional sex was suggested in the earliest HIV infection [29]. Given both injection and sexual risk associated with HIV outbreaks among PWID, there is potential for implementation of low-barrier PrEP initiation at places PWID frequently visit like SSPs.

Structural and social barriers to PrEP care impede access to groups at highest risk and limit its impact. As in our survey where 39.5% reported cost as a barrier, economic concerns are prominent barriers to care and patients are more likely to initially accept PrEP when it is offered directly and for free [11, 30]. Real and perceived barriers to PrEP care including cost, transportation, language issues, stigma, and immigration status can create disparities in PrEP engagement [31, 32] that particularly impact the drug-using community. Furthermore, PrEP is an understudied and underutilized HIV prevention strategy for PWID. The Bangkok Tenofovir Study showed a 48.9% decrease in HIV incidence in PWID who took daily tenofovir compared to those who took placebo, as well as significant interest among PWID in continuing PrEP after the trial [33, 34]. However, there has been a large gap in the literature about PrEP in PWID since that trial [14]. Our findings suggest limited interest in linkage to PrEP among PWID at the Miami SSP, and overwhelming barriers to PrEP use including low PrEP awareness and the same structural (e.g. stigma) and economic barriers (i.e. cost) that have been documented in MSM populations [31, 32]. Other studies support high acceptability of PrEP among PWID but note competing health priorities, including substance use disorders and need for treatment, as impediments to PrEP engagement [35].

Interestingly, in this study, the opioid use group had decreased odds of being interested in PrEP linkage. Our analysis by drug class showed that our polysubstance use class (opioid plus stimulant) exhibited behaviors associated with increased HIV risk (i.e. syringe sharing), and could explain why in addition to accessing the SSP this class of PWID had greater interest in PrEP linkage. Additionally, the polysubstance use PWID were more likely to be homeless and less likely to have Medicaid than the opioid use class, suggesting that low barrier PrEP access for PWID with increased risk of HIV infection should include wraparound services such as medication storage and case management for patient assistance programs or insurance qualification.

There are several limitations to our study. First, behavioral data relies on self-report and is subject to social desirability and recall biases. However, interviews were conducted with trusted SSP staff, in confidential settings to minimize these biases. Findings could have been strengthened by including questions on perceived risk during the interviews. Second, it is possible that since SSP team members were tasked with making appointments instead of dedicated and experienced PrEP navigators, that fewer PWID felt motivated to schedule appointments. Utilizing the SSP as a medical home with integrated, on-site PrEP, PWID would have decreased need for navigation while increasing initiation. Third, this data reflects participants at one newly established SSP in a city with relatively limited access to PrEP [36] and may not be generalizable to other cities. Despite these limitations, in cities late to adopt SSPs, on-site integration of PrEP as an upstream intervention may be helpful in increasing initiation among PWID.

Increasing access to PrEP among PWID is critical at this time when the President has declared a national emergency for the opioid crisis and when there have been reported HIV outbreaks in PWID in several American cities [20, 21, 37]. Whereas there was desire among 23% of the PWID surveyed to receive PrEP, the lack of any successful linkages suggests an urgent need to both increase knowledge of the effectiveness of PrEP in the PWID community as well as establish low barrier access to PrEP for PWID patients. Our study shows that cities late to adopt SSPs such as Miami could benefit from more comprehensive harm reduction and HIV prevention services.

## Conclusions

Knowledge, awareness, and interest in being linked to PrEP were low among PWID surveyed. In addition, through passive referral from the SSP, no study participants were successfully linked to a PrEP provider or received a PrEP prescription. Further research is needed to examine potential interventions to improve linkage to PrEP services among PWID, including low threshold services at community-based SSPs.

## Supporting information

S1 Dataset (XLSX)
